# Effects of Replacement of Dietary Fishmeal by Cottonseed Protein Concentrate on Growth Performance, Liver Health, and Intestinal Histology of Largemouth Bass (*Micropterus salmoides*)

**DOI:** 10.3389/fphys.2021.764987

**Published:** 2021-12-21

**Authors:** Yulong Liu, Qisheng Lu, Longwei Xi, Yulong Gong, Jingzhi Su, Dong Han, Zhimin Zhang, Haokun Liu, Junyan Jin, Yunxia Yang, Xiaoming Zhu, Shouqi Xie

**Affiliations:** ^1^State Key Laboratory of Freshwater Ecology and Biotechnology, Institute of Hydrobiology, Chinese Academy of Sciences, Wuhan, China; ^2^College of Advanced Agricultural Sciences, University of Chinese Academy of Sciences, Beijing, China; ^3^Hubei Engineering Research Center for Aquatic Animal Nutrition and Feed, Wuhan, China; ^4^The Innovative Academy of Seed Design, Chinese Academy of Sciences, Wuhan, China

**Keywords:** cottonseed protein concentrate, growth, inflammation, intestinal histology, TOR pathway

## Abstract

An 8-week feeding trial was conducted to explore the effects of replacement of dietary fishmeal by cottonseed protein concentrate (CPC) on growth performance, liver health, and intestine histology of largemouth bass. Four isoproteic and isolipidic diets were formulated to include 0, 111, 222, and 333 g/kg of CPC, corresponding to replace 0% (D1), 25% (D2), 50% (D3), and 75% (D4) of fishmeal. Two hundred and forty largemouth bass (15.11 ± 0.02 g) were randomly divided into four groups with three replicates per group. During the experiment, fish were fed to apparent satiation twice daily. Results indicated that CPC could replace up to 50% fishmeal in a diet for largemouth bass without significant adverse effects on growth performance. However, weight gain rate (WGR), specific growth rate (SGR), feed efficiency (FE), and condition factor (K) of the largemouth bass were significantly decreased when 75% of dietary fishmeal that was replaced by CPC. The whole body lipid content was increased with the increasing of dietary CPC levels. Oil red O staining results indicated that fish fed the D4 diet showed an aggravated fat deposition in the liver. Hepatocytes exhibited serious degeneration, volume shrinkage, and inflammatory cells infiltration in the D4 group. Intestinal villi appeared shorter and sparse with severe epithelial damage in the D4 group. The transcription levels of anti-inflammatory cytokines, such as transforming growth factor β (*tgf-*β), interleukin 10 (*il-10*), and interleukin 11 β (*il-11*β), were downregulated in the D4 group. The lipid metabolism-related genes carnitine palmitoyl transferase 1 (*cpt1*), peroxisome proliferator-activated receptor α (*ppar*α), and target of rapamycin (TOR) pathway were also significantly downregulated in the D4 group. It was concluded that suitable replacement of fishmeal by less than 222 g CPC/kg diet had a positive effect on growth performance of fish, but an excessive substitution of 75% fishmeal by CPC would lead to the suppressed growth, liver inflammation, and intestinal damage of largemouth bass.

## Introduction

Fishmeal is known as the preferential protein ingredient in aquafeeds due to its high digestible protein content, balanced amino acid profile, and fewer anti-nutritional factors (NRC, 2011). However, the rapid development of aquaculture and aquafeeds increases the imbalance between supply and demand of fishmeal because of the unsustainable fisheries resources and the increasing price of fishmeal (Naylor et al., 2009; Turchini et al., 2018). Therefore, finding new alternative and efficient fishmeal substitutes is becoming more and more concerning (Hardy, 2010; FAO, 2018). So far, plant proteins have been widely reported as the fishmeal substitute in aquafeeds, such as soybean meal (Lim and Dominy, 1990), rapeseed meal (Cheng et al., 2010), cottonseed meal (Lim, 2010), and peanut meal (Liu et al., 2012). However, due to the existence of anti-nutritional factors, unbalanced amino acid profile, and low feed availability, plant proteins often cause many negative impacts on different fishes (Glencross et al., 2020).

As an important plant protein, cottonseed meal has a relatively balanced amino acid profile and is often widely used as a fishmeal substitute. However, the presence of gossypol, a main anti-nutritional factor in cottonseed meals, brought many adverse effects on the growth and health of fish and severely limited its utilization in aquafeeds (Anderson et al., 2016; Wan et al., 2018). Thus, removing anti-nutritional factors from cottonseed meals will make it better use in aquafeeds. With the recent development of cottonseed processing, the cottonseed protein concentrate (CPC) was obtained by low-temperature extraction with low levels of anti-nutritional factors, such as extremely low gossypol levels (Liu et al., 2020). Therefore, compared to the traditional cottonseed meal, CPC is an excellent plant protein to replace fishmeal in aquafeeds. As a new type of non-grain protein source, CPC was mainly evaluated in replacing dietary fishmeal for the marine fish species in a few studies. It was reported that replacing fishmeal with 60% CPC did not show adverse effects on growth performance and intestinal health of juvenile golden pompano (*Trachinotus ovatus*) (Shen et al., 2020). A study in pearl gentian grouper (*Epinephelus fuscoguttatus* ♀ × *Epinephelus lanceolatu*♂) found that CPC replacing up to 24% of fishmeal exhibited no negative effects on the growth and intestinal morphology of the fish (Chen et al., 2020). It was also observed that dietary inclusion of CPC suppressed growth performance and immune function of hybrid grouper (*Epinephelus fuscoguttatus* ♀ × *Epinephelus lanceolatu*♂) (Yin et al., 2018). When fed with a full plant protein diet with soybean protein concentrate and CPC, Japanese seabass (*Lateolabrax japonicas*) was induced abnormal metabolism in the liver and a fatty liver (Zhang et al., 2019). However, very little information on replacing fishmeal with CPC has been published in freshwater fish species, especially carnivorous fish that usually need a large content of dietary fishmeal.

Largemouth bass (*Micropterus salmoides*), a typical freshwater carnivorous fish, is one of the most important commercial cultivated fish in China with an annual production of more than 0.6 million tons in 2020 (He et al., 2020; China Fishery Statistical Yearbook, 2021). The dietary protein requirement for largemouth bass is about 48%, and it strongly depends on fishmeal, usually up to 40–50% (Huang et al., 2017). Therefore, alternative low-cost protein sources for largemouth bass are important for reducing feed prices and increasing the income of fishermen (Zhao et al., 2021). Till now, only one recent study evaluated the combination use of CPC and poultry by-product meal for replacing fishmeal in largemouth bass and mainly focused on the effects of the supplementation with CPC on growth performance and environmental impacts (Wang et al., 2021), but the investigation of replacing fishmeal with CPC on liver health and intestinal health of largemouth bass, especially at high replacing levels, has not been reported. In the present study, the effects of graded levels of dietary CPC on growth performance, liver health, and intestinal histology were conducted in largemouth bass to evaluate the feasibility for CPC replacing of fishmeal.

## Materials and Methods

### Experimental Diets

All ingredients and proximate compositions of the experimental diets are shown in Table 1. Four isonitrogenous and isolipidic experimental diets were formulated to include 0, 111, 222, and 333 g/kg of CPC, corresponding to replace 0% (D1), 25% (D2), 50% (D3), and 75% (D4) of dietary fishmeal. The designed protein and lipid content of the experimental diets are based on Huang et al. (2017) and Li et al. (2020), which can satisfy the requirement of largemouth bass. D1 group was used as the control diet with 40% of fishmeal. All diets were processed through an extruder (TSE65S; Modern Yanggong Machinery S&T development CO., LTD., Beijing, China) and made into 2 mm diameter floating pellets. All diets were dried at 60°C in an oven and stored at 4°C. The composition of the CPC used in this experiment is shown in Table 2. Table 3 shows the amino acids profile of CPC and the experimental diets.

**TABLE 1:** Formulation and composition of the experimental diets for largemouth bass.

Ingredients (g/kg dry matter)	Diets^1^			
	D1	D2	D3	D4
Fish meal^2^	400	300	200	100
Blood meal^3^	40	40	40	40
Gluten^4^	50	50	50	50
Soybean meal^5^	100	100	100	100
Soybean protein concentrate^6^	130	130	130	130
Cottonseed protein concetrated^7^	0	111	222	333
Cassava starch^8^	110	110	110	110
Fish oil^9^	32.6	35.8	39.0	42.2
Soybean oil	32.6	35.8	39.0	42.2
Vitamin and mineral additives^10^	10	10	10	10
Monocalcium phosphate	15	15	15	15
Choline chloride	1	1	1	1
Microcrystalline cellulose	78.8	61.4	44.0	26.6
Proximate chemical compositions (g/kg dry matter)				
Moisture	21.1	21.0	23.7	20.2
Crude protein	504.3	498.3	497.4	505.4
Crude lipid	83.2	89.7	83.3	85.5
Free gossypol (mg/kg)	–	80	151	284

*^1^D1–D4: 0, 25, 50, and 75% of the fish meal was replaced by cottonseed protein concentrate.*

*^2^Fish meal: From Superprime, TASA Fish Product Co. Ltd, Peru.*

*^3^Blood meal: From Beijing Yangyuan Veterinary Medicine Technology Co., Ltd, Beijing, China.*

*^4^Gluten: From Henan Midaner Trading Co., Ltd, Xinzheng, Henan, China.*

*^5^Soybean meal: From Qingdao Bohai Agricultural Development Co., Ltd, Qingdao, China.*

*^6^Soybean protein concentrate: From Yihai grain and oil industry Co., Ltd, Taizhou, Jiangsu, China.*

*^7^Cottonseed protein concetrated: From Xinjiang Jinlan Plant Protein Co., Ltd, Xinjiang, China.*

*^8^Cassava starch: From Wuhan Yiteng Starch Co., Ltd, Wuhan, China.*

*^9^Fish oil: Peru anchovy oil, purchased from Coland Feed Co., Ltd., Wuhan, China.*

*^10^Vitamin and mineral additives: From Guangdong Nutriera Group, Guangzhou, China.*

**TABLE 2:** Proximate composition of cottonseed protein concentrate used in the experimental diets (g/kg dry matter).

Proximate composition	Cottonseed protein concentrate
Moisture	16.7
Crude protein	615.1
Crude lipid	23.6
Ash	81.2
Free gossypol (mg/kg)	709

**TABLE 3:** Amino acid composition of the experimental diets (g/kg dry matter).

	D1	D2	D3	D4	CPC
**Essential amino acids (EAAs)**					
Met	5.80	6.46	5.12	2.71	2.96
Lys	29.63	32.35	27.21	25.26	24.68
Thr	16.88	17.92	15.96	15.85	18.08
Ile	18.33	19.25	16.94	16.74	18.46
His	14.21	14.52	14.41	15.07	17.81
Val	19.94	19.87	19.60	20.26	22.95
Leu	35.78	37.23	34.07	33.78	34.08
Arg	19.42	17.16	20.91	23.96	41.85
Phe	22.52	21.71	22.52	24.45	33.33
**Non-essential amino acids (NEAAs)**					
Asp	37.09	37.25	36.47	37.41	44.87
Ser	19.06	19.13	18.80	19.89	24.16
Glu	70.77	68.74	73.14	84.61	95.16
Gly	22.14	23.15	20.91	20.25	24.54
Ala	23.17	24.39	21.25	20.34	22.56
Cys	1.78	0.48	1.53	2.67	3.83
Pro	24.47	23.71	22.83	23.37	24.03
Tyr	12.07	12.62	11.78	12.13	14.33

*D1–D4: 0, 25, 50, and 75% of the fish meal was replaced by cottonseed protein concentrate. CPC, cottonseed protein concentrate.*

### Fish and Feeding Trial

Largemouth bass was purchased from a commercial fish farm (Ezhou, Hubei, China). All fish were fed the control D1 diet for 2 weeks to acclimate the experimental condition. The experiment was conducted in an indoor recirculating system with 12 circular plastic tanks (volume 140 L). At the beginning of the experiment, all fish were deprived of feed for 24 h. Two hundred and forty healthy fish with an initial body weight of 15.11 ± 0.02 g were randomly divided into 4 groups with 3 replicates in each group and 20 fish per replicate.

During the experiment, fish were fed to apparent satiation twice daily at 08:30 and 16:30 for 8 weeks. Daily feed intake was recorded, and uneaten feed was taken out and recorded. This experiment was conducted under 12 h light: 12 h dark, and aeration was supplied to each tank 24 h per day. The water temperature was maintained at 27.0°C ± 1.0°C, pH 7–8, dissolved oxygen > 5.0 mg/L, and ammonia-N < 0.5 mg/L.

### Sampling

At the end of the feeding trial, all fish were deprived of diets for 24 h. Then, all fish in each tank were bulk weighted to calculate mean final body weight (FBW), weight gain rate (WGR), specific growth rate (SGR), and feed efficiency (FE). Two fish from each tank were randomly selected and frozen at − 20°C for the analysis of body composition. Six fish from each tank were anesthetized with 60 mg/L MS-222, fish body weight and length, viscera, and liver weight of the three fish were recorded to calculate condition factor (K), hepatosomatic index (HSI), and viscerosomatic index (VSI). Three fish from each tank were selected, and liver samples near to the bile were collected, fast-frozen in liquid nitrogen, and stored at − 80°C for mRNA expression, enzyme analysis, and western blot analysis. Another three fish from each tank were sampled, and the liver tissues were collected and stored at − 20°C to determine the fat content of the liver. The liver and mid-intestine tissues from the three fish per tank were sampled, approximately 5 mm × 5 mm × 5 mm tissues were fixed in 4% paraformaldehyde (Boerfu Biotechnology Co., Ltd., Wuhan, China) and stored at 4°C no longer than 48 h for histological analysis.

### Biochemical Assays

Analysis of the proximate composition of the experimental diets and fish samples was executed according to an AOAC protocol (Association of Official Analytical Chemists [AOAC], 2003). Moisture content was determined by oven at 105°C until constant weight. Ash content was measured by incineration in a muffle furnace (Muffle furnace, Yingshan, Hubei, China) at 550°C for 12 h. Crude protein content was measured by the Kjeldahl method using a Kjeltec analyzer unit (Foss Tecator, Höganäs, Sweden), and crude lipid content was measured by the Soxtec system (Soxtec System HT6, Tecator, Höganäs, Sweden). The amino acid contents of all experimental diets were determined after acid hydrolysis in 6 N HCl for 24 h at 11°C according to the method of Tu et al. (2015) and then were analyzed using Hitachi L-8800 Amino Acid Analyzer (Hitachi High-Technologies Corporation, Tokyo, Japan). Free gossypol levels of the experimental diets and CPC were determined according to the aniline method (Bian et al., 2016). Free gossypol was extracted in the presence of 3-amino-1-propanol with a mixture of 2-propanol and hexane. The extracted free gossypol was converted into gossypol-aniline with aniline. Finally, the absorbance of the compound was measured using a spectrophotometer at the wavelength of 440 nm. Liver samples were freeze-dried (Alpha 1-4 LD-plus, Christ, Osterode, Germany), and then the liver lipid content was determined by chloroform/methanol (V/V, 2:1) extraction method.

Activities of alkaline phosphatase (AKP), catalase (CAT), total superoxide dimustase (T-SOD), and malondialdehyde (MAD) content of liver tissues were measured according to the instructions of the commercial kits (Nanjing Jiancheng Bioengineering Institute, Nanjing, Jiangsu, China).

### Histological Analysis

The liver and mid-intestine tissues fixed by 4% paraformaldehyde were dehydrated in a series of ethanol, embedded in paraffin, and cut into 5 μm sections. The sections were stained following the protocols of hematoxylin and eosin (H&E). Frozen liver sections were fixed in formalin and stained with oil red O. Then the samples were observed and photographed by using a microscope system (DM1000, Leica Microsystems, Germany). The image was analyzed by Image-Pro Plus 6.0.

### Quantitative Real-Time PCR Analysis

Total RNA of the liver was extracted using TRIzol reagent and electrophoresed on an agarose gel to evaluate the integrity. The concentration of extracted RNA was spectrophotometrically quantified with Nanodrop 2000 (Thermo Fisher Scientific, Waltham, MA, United States). In total, 1 μg of extracted total RNA was reverse transcribed to cDNA using an M-MLV First-Strand Synthesis Kit (Invitrogen, Shanghai, China), and the obtained cDNA was used for PCR. The quantitative real-time PCR was performed in LightCycle^§^ 480 II system in a 6 μl reaction volume containing LightCycle 480 SYBR Green I Master Mix (Roche, Switzerland). The final reaction volume of 6 μl includes 2 μl cDNA, 0.24 μl forward and reverse primer, 0.52 μl ddH_2_O, and 3 μl LightCycle 480 SYBR Green I Master Mix. Negative controls were done in the same template which template was replaced with ddH_2_O. The qPCR was conducted with the following condition: 95°C for 5 min followed by 40 cycles with 10 s at 95°C, 20 s at Tm, and 10 s at 75°C. After PCR final cycle, the melted curve was performed to confirm the amplification of a single product with 0.5°C increment from 65°C to 95°C. β*-actin* and *ef-1*α, expressed very stable in largemouth bass liver tissue, were chosen as the endogenous reference for normalization. The primers used for transcriptional expression were obtained from previous literatures (Table 4). The results were calculated according to the method of Su et al. (2020).

**TABLE 4:** Primers used in quantitative real-time PCR analysis.

Gene name	Primers	Sequence 5′-3′	Sources
β*-actin*	F	ATCGCCGCACTGGTTGTTGAC	Chen et al., 2012
	R	CCTGTTGGCTTTGGGGTTC	
*ef-1*α	F	TGCTGCTGGTGTTGGTGAGTT	Yu et al., 2019
	R	TTCTGGCTGTAAGGGGGCTC	
*tgf-*β	F	GCTCAAAGAGAGCGAGGATG	Yu et al., 2019
	R	TCCTCTACCATTCGCAATCC	
*il-10*	F	CGGCACAGAAATCCCAGAGC	Yu et al., 2019
	R	CAGCAGGCTCACAAAATAAACATCT	
*il-11*β	F	TTCCCAACAGACAGATGAAGAACTC	Yu et al., 2019
	R	TGCCTGTGTTCAGCCAGTCAA	
*tnf-*α	F	CTTCGTCTACAGCCAGGCATCG	Yu et al., 2019
	R	TTTGGCACACCGACCTCACC	
*il-15*	F	GTATGCTGCTTCTGTGCCTGG	Yu et al., 2019
	R	AGCGTCAGATTTCTCAATGGTGT	
*cpt1*	F	CATGGAAAGCCAGCCTTTAG	Yu et al., 2019
	R	GAGCACCAGACACGCTAACA	
*ppar*α	F	CCACCGCAATGGTCGATATG	Yu et al., 2019
	R	TGCTGTTGATGGACTGGGAAA	
*Fasn*	F	TGTGGTGCTGAACTCTCTGG	Yu et al., 2019
	R	CATGCCTAGTGGGGAGTTGT	
*Tor*	F	TCAGGACCTCTTCTCATTGGC	Li et al., 2021
	R	CCTCTCCCACCATGTTTCTCT	
*s6*	F	GCCAATCTCAGCGTTCTCAAC	Li et al., 2021
	R	CTGCCTAACATCATCCTCCTT	

*F, forward primer; R, reverse primer; ef-1α, elongation factor 1α; tgf-β, transforming growth factor β; il-10, interleukin 10; il-11β, interleukin 11 β; tnf-α, tumor necrosis factor α; il-15, interleukin; cpt1, carnitine palmitoyl transferase 1; pparα, peroxisome proliferator-activated receptor α; tor, target of rapamycin; s6, ribosomal protein.*

### Western Blot Analysis

The western blot analysis was carried out according to the method described by Yang et al. (2018). Liver tissues were lysed by RIPA lysis buffer (Beyotime Biotechnology, China) with protease inhibitor cocktail and phosphatase inhibitor cocktail (Roche, Basel, Switzerland). Equal amounts of protein were separated on sodium dodecyl sulphate-polyacrylamide gel electrophoresis (SDS-PAGE) gels and transferred to poly(vinylidene fluoride) (PVDF) membranes. The PVDF membranes were blocked for 1 h with 5% milk in TBST buffer and then incubated with anti-phospho-m-target of rapamycin (TOR) (1:1000, #2971; CST, Danvers, MA, United States), anti-mTOR Antibody (1:1000, #2972; CST, Danvers, MA, United States), anti-phospho-ribosomal protein (S6)^*Ser*235/236^ (1:1000, #4858; CST, Danvers, MA, United States), anti-S6 (1:1000, #2217; CST, Danvers, MA, United States), and anti-GAPDH (1:1000, ab8245; Abcam). Horseradish peroxidase-labeled secondary antibodies were used to generate a chemiluminescent signal that was detected by ImageQuant LAS 4000mini (GE Healthcare Life Sciences). GAPDH was used as a control.

### Calculation and Statistical Analysis

The WGR, SGR, FE, K, VSI, and HSI were calculated as follows:


WGR(%)=100×(FBW-initialbodyweight)/initialbodyweight.

SGR(%/d)=100×[LnFBW-LnIBW]/days.

FE(%)=100×bodyweightgain/dryfeedconsumed.

K(g/cm)3=100×FBW/(bodylength).3

VSI(%)=100×visceraweight/wholebodyweight.

HSI(%)=100×liverweight/wholebodyweight.

All data were analyzed by SPSS 20 (SPSS Inc., Chicago, IL, United States) for windows. Before one-way ANOVA tested the differences among groups, all data were tested normal distribution and homogeneity of variances. After ANOVA identified the differences, Duncan’s multiple range testing was used to identify the significant differences between group means. Effects with a probability of *P* < 0.05 were considered statistically significant. All data are presented as means ± SE.

## Results

### Growth Performance

Effects of replacement of fishmeal with CPC on growth performance, feed utilization, and morphological indices of largemouth bass are shown in Table 5. The WGR in the D4 group was significantly lower (*P* < 0.05) than other groups, and there was no significant difference between D2 or D3 and D1 group (*P* > 0.05). And D4 group had the lowest SGR than other groups. Similar results were found in SGR and FE (*P* < 0.05). In addition, compared to the other groups, replacing fishmeal with CPC by 75% (D4 group) significantly decreased (*P* < 0.05) K and HSI of largemouth bass. But no significant differences were observed in VSI of largemouth bass fed different CPC diets (Table 5).

**TABLE 5:** The growth performance of largemouth bass fed different cottonseed protein concentrate diets for 8 weeks.

Items	Groups			
	D1	D2	D3	D4
IBW (g)	15.2 ± 0.09	15.1 ± 0.02	15.0 ± 0.02	15.2 ± 0.02
FBW (g)	63.1 ± 1.1^b^	63.1 ± 1.3^b^	63.5 ± 0.01^b^	54.9 ± 2.0^a^
WGR (%)	316.4 ± 7.6^b^	317.4 ± 8.4^b^	322.0 ± 0.6^b^	261.8 ± 12.6^a^
SGR (%/d)	2.7 ± 0.03^b^	2.7 ± 0.04^b^	2.70 ± 0.003^b^	2.4 ± 0.07^a^
FE (%)	119.7 ± 1.0^b^	119.9 ± 0.4^b^	118.8 ± 0.7^b^	107.2 ± 2.1^a^
K (g/cm^3^)	2.2 ± 0.02^b^	2.2 ± 0.01^b^	2.2 ± 0.03^b^	2.1 ± 0.05^a^
VSI (%)	8.1 ± 0.2	8.4 ± 0.3	8.1 ± 0.2	8.5 ± 0.5
HSI (%)	2.9 ± 0.2^b^	3.0 ± 0.2^b^	2.7 ± 0.2*^ab^*	2.3 ± 0.2^a^

*The results are presented as means ± SE values in the same column with different superscripts are significantly different (P < 0.05).*

### Body Composition

A significant increase (*P* < 0.05) of body lipid content was observed in the D4 group compared to the control D1 group, but no significant change (*P* > 0.05) of body lipid content was found between the D2 or D3 and D1 groups (Table 6). No changes of crude protein and moisture contents (*P* > 0.05) were found in largemouth bass fed different CPC diets. D4 group had a lower ash content (*P* < 0.05) than D1 and D2 groups (Table 6).

**TABLE 6:** Effects of replacing fish meal with cottonseed protein concentrated on body composition of largemouth bass.

Items	Groups			
	D1	D2	D3	D4
Crude protein (%)	17.19 ± 0.12	17.29 ± 0.15	17.24 ± 0.10	16.98 ± 0.10
Crude lipid (%)	5.94 ± 0.29^a^	6.34 ± 0.21*^ab^*	6.86 ± 0.43*^ab^*	7.16 ± 0.33^b^
Ash (%)	3.71 ± 0.07^b^	3.85 ± 0.24^b^	3.68 ± 0.06*^ab^*	3.39 ± 0.06^a^
Moisture (%)	72.37 ± 0.42	71.80 ± 0.14	71.63 ± 0.35	71.95 ± 0.40

*The results are presented as means ± SE values in the same column with different superscripts were significantly different (P < 0.05).*

**FIGURE 1:**
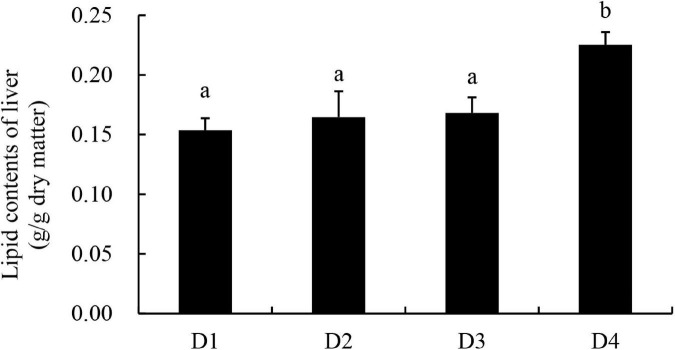
Lipid content of liver tissues in largemouth bass fed different cottonseed protein concentrate diets for 8 weeks. Bars assigned with different letters were significantly different (*P* < 0.05).

Similar to the results of body lipid content, the liver lipid content of largemouth bass increased with increasing dietary CPC levels, and the D4 group exhibited a significantly higher (*P* < 0.05) content of liver lipid than other groups (Figure 1).

### Histomorphology

**FIGURE 2:**
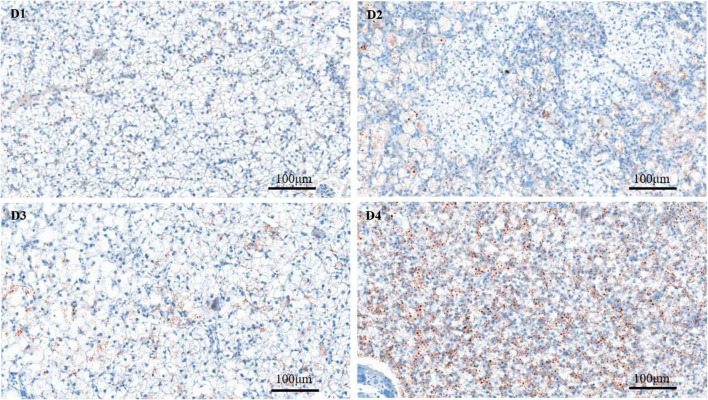
Oil Red O staining (200×) of fat deposition of live tissues in largemouth bass fed different cottonseed protein concentrate diets for 8 weeks. Red points represent lipid droplets. Scale bar: 100 μm. D1–D4: 0, 25, 50, and 75% of the fish meal was replaced by cottonseed protein concentrate.

**FIGURE 3:**
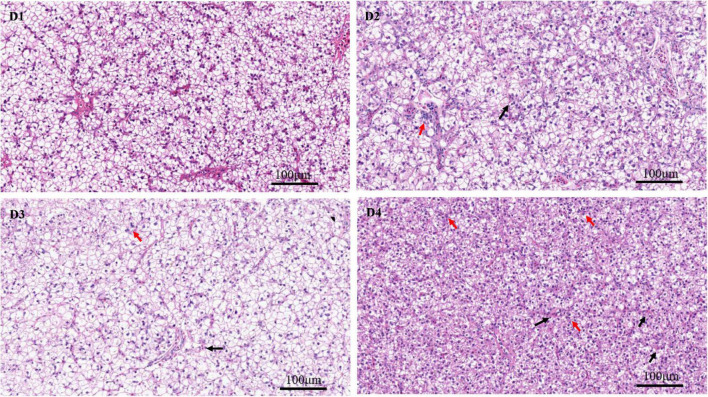
H & E staining (200×) of liver histomorphology in largemouth bass fed different cottonseed protein concentrate diets for 8 weeks (black arrows show the inflammatory cells infiltration, and red arrows show the nucleus pyknosis). Scale bar: 100 μm. D1–D4: 0, 25, 50, and 75% of the fish meal was replaced by cottonseed protein concentrate.

Through the oil red O staining of the liver, it was found that fish in the D4 group showed an aggravated fat deposition (Figure 2). The hepatocytes in fish fed the control D1 diet showed clear boundary with vacuolated cytoplasm and no obvious vacuolar degeneration and inflammatory cells infiltration (Figure 3). However, with the increasing dietary CPC levels, largemouth bass a exhibited different degree of hepatocyte degeneration and inflammatory cells infiltration. Compared to the control D1 group, the hepatocytes of fish in the D4 group exhibited severe abnormalities with a disappeared boundary of hepatocytes, hepatocytes volume shrinkage, nucleus pyknosis, aggravated vacuolar degeneration, and inflammatory cells infiltration (Figure 3).

**FIGURE 4:**
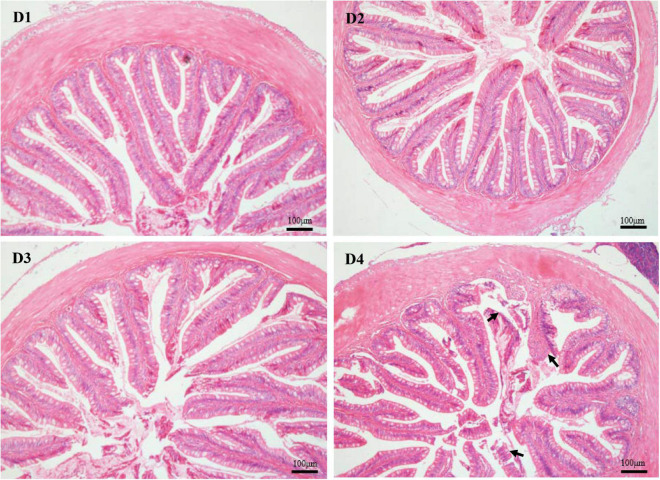
H & E staining (100×) of mid-intestinal histology in largemouth bass fed different cottonseed protein concentrate diets for 8 weeks (black arrows show the epithelial damage). Scale bar: 100 μm. D1–D4: 0, 25, 50, and 75% of the fish meal was replaced by cottonseed protein concentrate.

As shown in Figure 4, the structure of the mid-intestine in the D1, D2, or D3 group is normal. However, when 75% of dietary fishmeal was replaced by CPC, the intestinal villi of largemouth bass appeared shorter and sparse with severe epithelial damage in the D4 group. D4 group exhibited significantly lower (*P* < 0.05) villus height and width than the D1 group (Table 7).

**TABLE 7:** Histological parameters of mid-intestinal in largemouth bass fed different cottonseed protein concentrate diets.

Groups	Villus height (μm)	Villus width (μm)
D1	564.43 ± 20.67^b^	110.81 ± 3.89^b^
D2	566.98 ± 15.60^b^	99.93 ± 6.01^ab^
D3	519.32 ± 13.41a^b^	99.99 ± 2.45^ab^
D4	476.63 ± 8.48^a^	96.12 ± 3.52^a^

*The results are presented as means ± SE values in the same column with different superscripts were significantly different (P < 0.05).*

### Liver Antioxidant Enzymes

**FIGURE 5:**
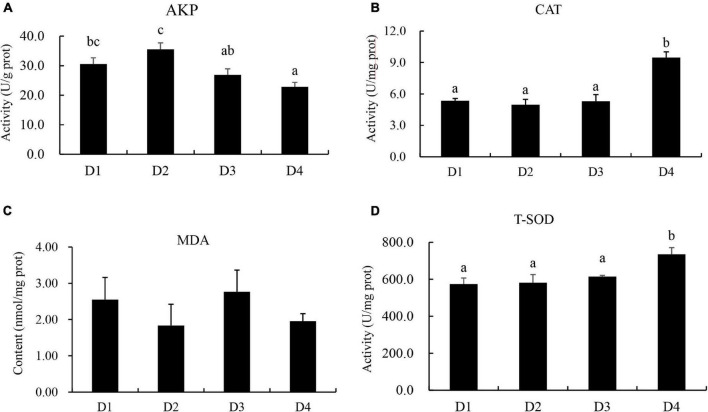
Effects of replacing fish meal with cottonseed protein concentrate on liver antioxidant capacities of largemouth bass. **(A)** AKP, alkaline phosphatase. **(B)** CAT, catalase. **(C)** MDA, malondialdehyde. **(D)** T-SOD, total superoxide dimustase. Bars assigned with different letters were significantly different (*P* < 0.05).

Fish in the D2 group had the highest activity of liver AKP, and the D4 group had the lowest activity of AKP among all groups (Figure 5). The activities of CAT and T-SOD in the D4 group were significantly higher (*P* < 0.05) than other groups. No significant difference in the MDA content was found (*P* > 0.05) in largemouth bass fed different CPC diets (Figure 5).

### Gene Expression and Protein Level in Liver Tissues

**FIGURE 6:**
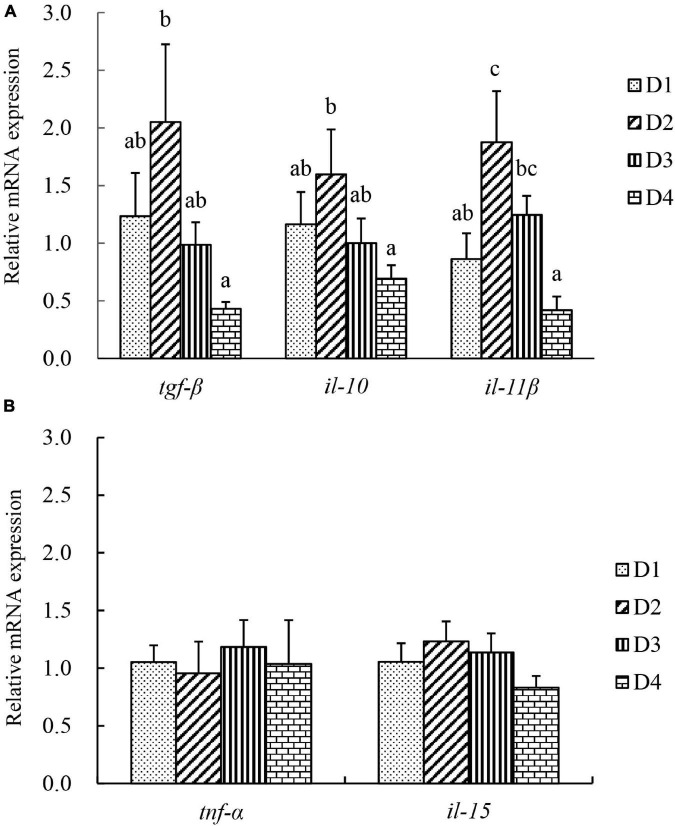
The transcriptional level of inflammation-related genes in liver of largemouth bass fed different cottonseed protein concentrate diets for 8 weeks. **(A)** Anti-inflammatory related cytokines, transforming growth factor β (*tgf-*β), interleukin 10 (*il-10*), and interleukin 11β (*il-11*β). **(B)** Pro-inflammatory related cytokines, tumor necrosis factor α (*tnf-*α), and interleukin (*il-15*). Bars assigned with different letters were significantly different (*P* < 0.05).

The transcriptional level of inflammation-related genes in liver tissues is shown in Figure 6. Anti-inflammatory-related cytokines transforming growth factor β (*tgf-*β), interleukin 10 (*il-10*), and interleukin 11 β (*il-11*β) were significantly induced in the D2 group, but were significantly downregulated in the D4 group (*P* < 0.05). However, the mRNA expression of pro-inflammatory cytokines *tnf-*α and *il-15* was not affected by the replacement of fishmeal by CPC (*P* > 0.05).

**FIGURE 7:**
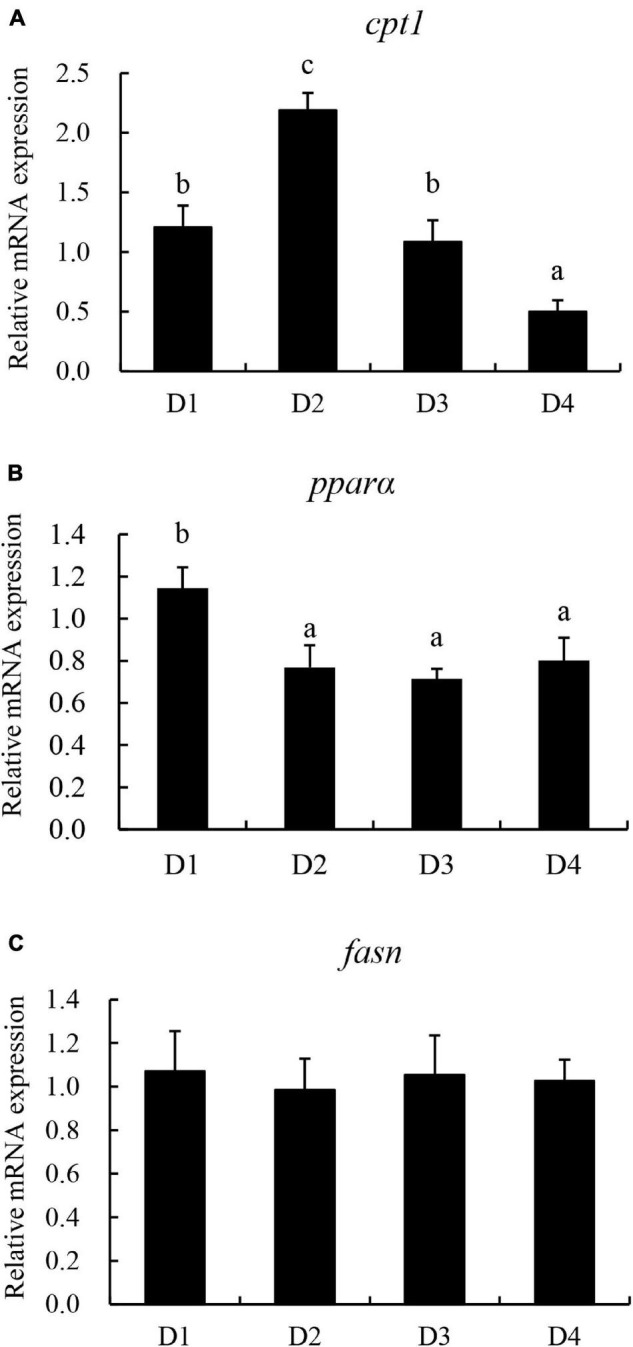
Effects of replacing fishmeal with cottonseed protein concentrate on the transcriptional expression of lipid metabolism-related genes in liver of largemouth bass. **(A)** Carnitine palmitoyl transferase 1 (*cpt1*). **(B)** Peroxisome proliferator-activated receptor α (*ppar*α). **(C)** Fatty acid synthase (*fasn*). Bars assigned with different letters were significantly different (*P* < 0.05).

The relative expression of lipid metabolism-related genes in the liver is presented in Figure 7. The genes related to lipolysis peroxisome proliferator-activated receptor α (*ppar*α) and carnitine palmitoyl transferase 1 (*cpt1*) were downregulated with the increasing dietary CPC levels. The transcriptional level of *cpt1* was significantly higher (*P* < 0.05) in the D2 group than in the other groups. However, the relative expression of *fasn* was not significantly affected (*P* > 0.05) by the replacing of fishmeal with CPC.

**FIGURE 8:**
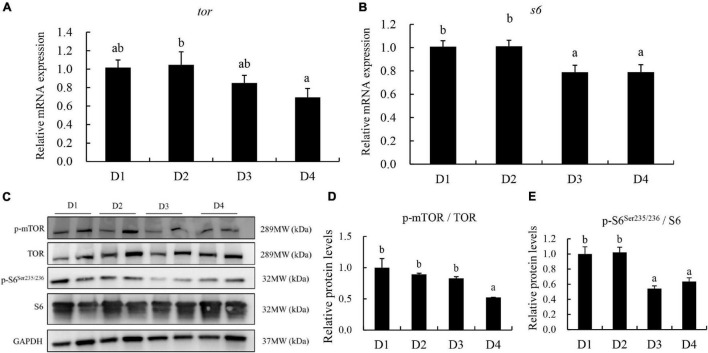
The transcriptional and protein level of TOR and S6 in the liver of largemouth bass fed different cottonseed protein concentrate diets for 8 weeks. **(A)** Relative mRNA expression of target of rapamycin (*tor*). **(B)** Relative mRNA expression of ribosomal protein (*s6*). **(C–E)** Western blot analysis for protein levels of *tor* and *s6*. Bars assigned with different letters were significantly different (*P* < 0.05). D1–D4: 0, 25, 50, and 75% of the fish meal was replaced by cottonseed protein concentrate.

The relative expression of *TOR* and *S6*was downregulated in fish with increasing dietary CPC levels (Figures 8A,B). Compared to the control D1 group, the D2 group did not exhibit a negative regulation (*P* > 0.05) of gene expression of *TOR* and *S6*. However, D4 group had the lowest level of relative expression of *tor* among all groups (Figures 8A,B). The corresponding protein levels of TOR and S6 were also detected. The phosphorylation activation of TOR and S6 was significantly downregulated (*P* < 0.05) in the D4 group (Figures 8C–E).

## Discussion

Many studies have been conducted to investigate the effects of replacing dietary fishmeal with plant protein on the growth performance of fish (Yin et al., 2018; Abbasi et al., 2020; Shen et al., 2020; Yaghoubi et al., 2020). In the present study, dietary replacement of 25% or 50% of dietary fishmeal by CPC had no negative effects on growth performance, but dietary substitution of 75% fishmeal by CPC significantly decreased the growth performance of the fish. The present results were supported by previous results that no adverse effects on growth performance were found in juvenile golden pompano when dietary fishmeal was replaced by CPC at a moderate level, but high substitution led to a significant negative effect on the growth performance of fish (Shen et al., 2020). It was also reported that in hybrid grouper, WGR and SGR were significantly declined when over 48% of dietary fishmeal was replaced by CPC (Yin et al., 2018). These findings indicated that a suitable replacement level of CPC in the diet showed no negative effects and even had positive effects on growth performance. It has been reported that a suitable CPC level in the diet may improve the amino acids profile in feed and make the proportion of amino acids more reasonable and suitable for aquatic animals (Elangovan and Shim, 2000; Yin et al., 2018). However, the high incorporation of CPC in diets impaired the growth performance of largemouth bass. Dietary CPC inclusion was often accompanied by poor palatability, imbalanced amino acid composition, and anti-nutritional factors (Chen et al., 2020; He et al., 2021). When replacing fishmeal with a high level, it would significantly affect the growth of fish (Yin et al., 2018; He et al., 2021). Our research results demonstrated that 111–222 g/kg CPC is the suitable replacement level for largemouth bass.

The liver is the most important organ of body metabolism (Tamura and Shimomura, 2005). In the present study, the lipid deposition in liver tissues of largemouth bass was significantly increased in the D4 group. Similar results were reported that there was obvious fat deposition in liver tissues of hybrid grouper fed diet with the replacement of 36% fishmeal by CPC (Yin et al., 2018). Zhang et al. (2019) also reported that fatty liver was induced in Japanese seabass fed a full plant protein diet of soybean protein and CPC which was explained that a full plant protein diet caused nutrient and energy metabolic disorder and then induced fatty liver. In this study, 75% CPC replacement level might induce metabolic disorder in the liver. This was supported by the lipid metabolism-related gene expression (*cpt1*, *ppar*α, and *fasn*) and histological results. In the present study, the D4 group with replacement of 75% fishmeal by CPC had significant downregulation of TOR signaling, lipolysis-related genes, and anti-inflammatory cytokine genes, which indicated that the metabolism of fish was significantly affected by the high substitution of CPC. In addition, from the histomorphology of the liver, we found that compared to the control D1 group, the hepatocytes of largemouth bass fed diet with the replacement of 75% fishmeal by CPC exhibited serious degeneration, volume shrinkage, and inflammatory cells infiltration. The results were well agreed with a previous study that juvenile turbot fed diet with 45% of fishmeal replaced by cottonseed meal led to smaller liver cell and liver damage (Bian et al., 2016). From the mid-intestinal histology, abnormal shorter and sparse with severe epithelial damage in fish was observed in the D4 group. Consistently, many researchers have reported that high plant proteins inclusion diet-induced intestinal structure damage in juvenile turbot (Bian et al., 2016), Japanese seabass (Wang et al., 2016), and largemouth bass (He et al., 2020). Therefore, all these indicated that 75% of dietary fishmeal replaced by CPC might induce abnormal liver fat deposition, liver, and intestinal damage of largemouth bass which would be closely related to the decreased growth of the fish.

Transforming growth factor β, il-10, and il-11β are the anti-inflammatory cytokines, and tnf-α and il-15 are two important pro-inflammatory cytokines, which play an important role in inflammatory processes and immune response to protect the liver from cell injury and initiate tissue regeneration (Yu et al., 2019; Zhang et al., 2019). In the present study, the gene expressions of *tgf-*β, *il-10*, and *il-11*β were significantly downregulated with the increasing dietary CPC levels, and these genes exhibited the highest transcriptional levels in the D2 group. Anti-inflammatory cytokines can prevent abnormal expression of immune response (Hoseinifar et al., 2018). The present results observed upregulation of anti-inflammatory cytokines in the D2 group (substitution of 25% fishmeal) and downregulation of anti-inflammatory cytokines in the D4 group (substitution of 75% fishmeal), together with the no changes of pro-inflammatory cytokines among all groups. All these indicated that low substitution of fishmeal by CPC caused an improved immune response of largemouth bass, but excessive substitution led to a significant decline in immune status which would also be closely related to the decreased growth and tissue damage of the fish.

In mammals and fish, the TOR signaling pathway, such as *tor*, *s6*, and so on, plays a key role in sensing nutrients and regulates organismal growth and homeostasis by coordinating the anabolism and catabolism (Lansard et al., 2010; Roux and Topisirovic, 2012; Bian et al., 2017). Furthermore, there is a positive correlation between the phosphorylation activation of the TOR pathway and growth in fish (Song et al., 2016; Bian et al., 2017). In the present study, the transcriptional and protein levels of *tor* and *s6* were significantly downregulated in fish of the D4 group (high substitution of 75% fishmeal). Similar results were reported by Zhou et al. (2018) that high dietary inclusion of plant protein decreased the relative expression of TOR pathway-related genes. Some studies have shown that gossypol and imbalanced amino acids can suppress TOR signaling (Tu et al., 2015; Bian et al., 2017). In the present study, high dietary inclusion of CPC contained high gossypol and imbalanced amino acids, which negatively regulated the TOR pathway. This indicated that the impaired TOR signaling pathway is one reason for the negative effect on growth performance in largemouth bass fed D4 diet.

In this study, the content of whole body protein and moisture were not affected by different dietary CPC inclusion. Similar results were reported in *Oncorhynchus mykiss* (Zhao et al., 2021) and *Litopenaeus vannamei* (Wan et al., 2018), which found that whole body protein and moisture contents were not significantly changed among CPC substitution groups. In the present study, whole body lipid content of largemouth bass was increased with the increasing dietary CPC levels. The result was consistent with previous results that the replacement of fishmeal by low-gossypol cottonseed meal increased whole body lipid content of juvenile southern flounder (*Paralichthys lethostigma*) and high dietary inclusion of low-gossypol cottonseed meal impaired the liver function of the fish (Alam et al., 2018). The present results of the increase of lipid content in whole body and liver tissues of fish with the increasing dietary CPC levels could be due to the significant downregulation of lipolysis genes and no change of lipogenesis in fish fed diets with high substitutions by CPC.

Compared to fishmeal, plant proteins are characterized as a high carbohydrate source (Zhang et al., 2019). High dietary carbohydrates often caused lipid metabolism disorder and caused lipid deposition in the liver (Zhang et al., 2020). In the current study, high dietary CPC along with high carbohydrates maybe one important reason accounting for liver lipid deposition in largemouth bass. pparα is one of the important transcriptional factors in regulating fatty acids oxidation and lipolysis (Goto et al., 2011). Similarly, cpt1 plays an important role in mediating long-chain fatty acids oxidation (Kerner and Hoppel, 2000). In the present study, the gene expression of *ppar*α and *cpt1* were lower in the D4 group than the D1 group. A similar result was reported that juvenile hybrid grouper fed a high level of dietary mixture plant protein downregulated pparα and cpt1 mRNA expression (Ye et al., 2019). fasn is an important enzyme involved in lipid synthesis, the mRNA level of *fasn* was not affected by different CPC inclusion level. The current results indicated that high-level dietary CPC inclusion caused lipid deposition in the liver of largemouth bass.

The organism antioxidant enzyme activity is an important indicator in reflecting the status of oxidative stress in fish response to external stimuli (Deng et al., 2015). T-SOD and CAT activities can reflect the ability of the body against oxidative stress (Yuan et al., 2019). In the present study, T-SOD and CAT were significantly increased in largemouth bass fed D4 diet, indicating that largemouth bass had suffered from oxidative stress caused by high lever CPC replacement, increased T-SOD and CAT activities moderated the damage caused by oxidative stress. Similar results were obtained in hybrid grouper (Yin et al., 2018). MDA is one of the important indicators of lipid peroxidation, it has a strong biotoxicity to cells (Parvez and Raisuddin, 2005). In the present study, there was no significant difference in MDA content among groups, which indicated that increased T-SOD and CAT mitigated the lipid peroxidation caused by oxidative stress. Similar results were observed in juvenile *Trachinotus ovatus* and *Penaeus monodon*, the MDA content in liver was not influenced by different CPC replacement level (Shen et al., 2020; Jiang et al., 2021). AKP is an important enzyme reflecting the health of organism, has a potential protective effect on fish (Ghehdarijani et al., 2016). In the current study, D2 group showed the highest AKP activity, but the D4 group had the lowest AKP activity among all groups. The present results indicated that oxidative stress occurred when 75% of dietary fishmeal was replaced by CPC, which may be one of the reasons for the negative effect on growth performance in largemouth bass fed high-level CPC diets.

## Conclusion

In the present study, CPC can replace up to 50% of dietary fishmeal without any adverse influence on the growth, body composition, antioxidant indices, and intestinal health of largemouth bass. However, high dietary inclusion of CPC (75% replacement of fishmeal) would cause significant negative effects on the growth performance, liver, and intestine health of the fish. The present results indicated that suitable substitution of fishmeal by less than 222g CPC/kg diet had a positive effect on growth performance of fish, but an excessive substitution of 75% fishmeal by CPC led to the suppressed growth, liver, and intestinal damage of largemouth bass.

## Data Availability Statement

The original contributions presented in the study are included in the article/supplementary material, further inquiries can be directed to the corresponding author.

## Ethics Statement

The animal study was reviewed and approved by the Institute of Hydrobiology, Chinese Academy of Sciences.

## Author Contributions

DH and YL designed the study. YL performed the experimental work and wrote the manuscript under the direction of DH. QL and LX contributed to perform the experiment. YY helped with biochemical analysis. JS, YG, ZZ, and HL gave helps for analyzing data. JJ, XZ, and SX provided suggestions on experimental design and manuscript writing. All authors contributed to the article and approved the submitted version.

## Conflict of Interest

The authors declare that the research was conducted in the absence of any commercial or financial relationships that could be construed as a potential conflict of interest.

## Publisher’s Note

All claims expressed in this article are solely those of the authors and do not necessarily represent those of their affiliated organizations, or those of the publisher, the editors and the reviewers. Any product that may be evaluated in this article, or claim that may be made by its manufacturer, is not guaranteed or endorsed by the publisher.

## References

[B1] Abbasi A. Oujifard A. Torfi M. M. Habibi H. Nafisi B. M. (2020). Dietary simultaneous replacement of fish meal and fish oil with blends of plant proteins and vegetable oils in yellowfin seabream (*Acanthopagrus latus*) fry: Growth, digestive enzymes, antioxidant status and skin mucosal immunity. *Aquacult. Nutr*. 26 1131–1111. 10.1111/anu.13070

[B2] Alam M. S. Watanabe W. O. Carroll P. M. Gabel J. E. Corum M. A. (2018). Evaluation of genetically-improved (glandless) and genetically-modified low-gossypol cottonseed meal as alternative protein sources in the diet of juvenile southern flounder *Paralichthys lethostigma* reared in a recirculating aquaculture system. *Aquaculture* 489 36–45.

[B3] Anderson A. D. Alam M. S. Watanabe W. O. Carroll P. M. Wedegaertner T. C. Dowd M. K. (2016). Full replacement of menhaden fish meal protein by low-gossypol cottonseed flour protein in the diet of juvenile black sea bass *Centropristis striata*. *Aquaculture* 464 618–628.

[B4] Association of Official Analytical Chemists [AOAC] (2003). *Official methods of analysis.* Rockville, MD: AOAC.

[B5] Bian F. Zhou H. He G. Wang C. (2016). Effects of replacing fishmeal with different cottonseed meals on growth, feed utilization, haematological indexes, intestinal and liver morphology of juvenile turbot (*Scophthalmus maximus* L.). *Aquacult. Nutr*. 23 1429–1439. 10.1111/anu.12518

[B6] Bian F. Y. Jiang H. W. Man M. S. Mai K. S. Zhou H. H. Xu W. (2017). Dietary gossypol suppressed postprandial TOR signaling and elevated ER stress pathways in turbot (*Scophthalmus maximus* L.). *Am. J. Physiol.: Endocrinol. Metab.* 312 E37–E47. 10.1152/ajpendo.00285.2016 27894064

[B7] Chen G. F. Yin B. Liu H. Y. Tan B. P. (2020). Effects of fishmeal replacement with cottonseed protein concentrate on growth, digestive proteinase, intestinal morphology and microflora in pearl gentian grouper (*Epinephelus fuscoguttatus* ♀× *Epinephelus lanceolatu*♂). *Aquacult. Res*. 51 2870–2884.

[B8] Chen Y. J. Liu Y. J. Yang H. J. Yuan Y. Liu F. J. Tian L. X. (2012). Effect of dietary oxidized fish oil on growth performance, body composition, antioxidant defense mechanism and liver histology of juvenile largemouth bass *Micropterus salmoides*. *Aquacult. Nutr*. 18 321–331. 10.1111/j.1365-2095.2011.00900.x

[B9] Cheng Z. Y. Ai Q. H. Mai K. S. Xu W. Ma H. M. Li Y. (2010). Effects of dietary canola meal on growth performance, digestion and metabolism of Japanese seabass. Lateolabrax japonicus. *Aquaculture*. 305 102–108. 10.1016/j.aquaculture.2010.03.031

[B10] China Fishery Statistical Yearbook (2021). *Fishery Bureau.* Beijing: Ministry of Agriculture, China Agriculture Press.

[B11] Deng J. M. Mai K. S. Chen L. Q. Mi H. F. Zhang L. (2015). Effects of replacing soybean meal with rubber seed meal on growth, antioxidant capacity, non-specific immune response, and resistance to *Aeromonas hydrophila* in tilapia (*Oreochromis niloticus* × *O. aureus*). *Fish Shellfish Immunol*. 44 436–444. 10.1016/j.fsi.2015.03.018 25804486

[B12] Elangovan A. Shim K. F. (2000). The influence of replacing fish meal partially in the diet with soybean meal on growth and body composition of juvenile tin foil barb (*Barbodes altus*). *Aquaculture* 189 133–144. 10.1016/S0044-8486(00)00365-3

[B13] FAO (2018). *The State of World Fisheries and Aquaculture 2018: Meeting the Sustainable Development Goals.* Rome: FAO.

[B14] Ghehdarijani M. S. Hajimoradloo A. Ghorbani R. Roohi Z. (2016). The effects of garlic-supplemented diets on skin mucosal immune responses, stress resistance and growth performance of the caspian roach (*Rutilus rutilus*) fry. *Fish Shellfish Immunol*. 49 79–83. 10.1016/j.fsi.2015.12.021 26700174

[B15] Glencross B. D. Baily J. Berntssen M. H. Hardy R. MacKenzie S. Tocher D. R. (2020). Risk assessment of the use of alternative animal and plant raw material resources in aquaculture feeds. *Rev. Aquacult*. 12 703–758. 10.1111/raq.12347

[B16] Goto T. Lee J. Teraminami A. Kim Y. Hirai S. Uemura T. (2011). Activation of peroxisome proliferator-activated receptor-alpha stimulates both differentiation and fatty acid oxidation in adipocytes. *J. Lipid Res*. 52 873–884. 10.1194/jlr.M011320 21324916 PMC3073464

[B17] Hardy R. W. (2010). Utilization of plant proteins in fish diets: Effects of global demand and supplies of fishmeal. *Aquacult. Res*. 41 770–776. 10.1111/j.1365-2109.2009.02349.x

[B18] He M. Li X. Q. Poolsawat L. Guo Z. H. Yao W. X. Zhang C. Y. (2020). Effects of fish meal replaced by fermented soybean meal on growth performance, intestinal histology and microbiota of largemouth bass (*Micropterus salmoides*). *Aquacult. Nutr*. 26 1058–1071. 10.1111/anu.13064

[B19] He Y. F. Guo X. W. Tan B. P. Dong X. H. Chi S. Y. (2021). Replacing fishmeal with cottonseed protein concentrate in feed for pearl gentian groupers (*Epinephelus fuscoguttatus* ♀× *E. lanceolatus* ♂): Effects on growth and expressions of key genes involved in appetite and hepatic glucose and lipid metabolism. *Aquacult. Rep*. 20:100710. 10.1016/j.aqrep.2021.100710

[B20] Hoseinifar S. H. Zou H. K. Doan H. V. Miandare H. K. Hoseini S. M. (2018). Evaluation of some intestinal cytokines genes expression and serum innate immune parameters in common carp (*Cyprinus carpio*) fed dietary loquat (*Eriobotrya japonica*) leaf extract. *Aquacult. Res*. 49 120–127. 10.1111/are.13440

[B21] Huang D. Wu Y. B. Lin Y. Y. Chen J. M. Karrow N. Ren X. (2017). Dietary protein and lipid requirements for juvenile largemouth bass, *Micropterus salmoides*. *J. World Aquacult. Soc*. 48 782–790. 10.1111/jwas.12417

[B22] Jiang S. Chen Z. B. Zhou F. L. Yang Q. B. Huang J. H. Yang L. S. (2021). Study on partial replacement of fish meal with concentrated dephenolized cottonseed protein in feed of *Penaeus monodon*. *Aquacult. Res*. 52 3871–3881. 10.1111/are.15231

[B23] Kerner J. Hoppel C. (2000). Fatty acid import into mitochondria. *BBA-Mol Cell Biol L*. 1486 1–17. 10.1016/S1388-1981(00)00044-5 10856709

[B24] Lansard M. Panserat S. Plagnes-Juan E. Seiliez I. Skiba-Cassy S. (2010). Integration of insulin and amino acid signals that regulate hepatic metabolism-related gene expression in rainbow trout: role of tor. *Amino Acids* 39 801–810. 10.1007/s00726-010-0533-3 20213441

[B25] Li S. L. Dai M. Qiu H. J. Chen N. S. (2021). Effects of fishmeal replacement with composite mixture of shrimp hydrolysate and plant proteins on growth performance, feed utilization, and target of rapamycin pathway in largemouth bass, *Micropterus salmoides*. *Aquaculture* 533:736185. 10.1016/j.aquaculture.2020.736185

[B26] Li X. Y. Zheng S. X. Ma X. K. Cheng K. M. Wu G. Y. (2020). Effects of dietary protein and lipid levels on the growth performance, feed utilization, and liver histology of largemouth bass (*Micropterus salmoides*). *Amino Acids* 52 1043–1061. 10.1007/s00726-020-02874-9 32683495

[B27] Lim C. (2010). Substitution of cottonseed meal for marine animal protein in diets for *Penaeus vannamei*. *J. World Aquacult. Soc*. 27 402–409.

[B28] Lim C. Dominy W. (1990). Evaluation of soybean meal as a replacement for marine animal protein in diets for shrimp (*Penaeus vannamei*). *Aquaculture* 87 53–63. 10.1016/0044-8486(90)90210-E

[B29] Liu H. Dong X. H. Tan B. P. Du T. Zhang S. Yang Y. Z. (2020). Effects of fish meal replacement by low-gossypol cottonseed meal on growth performance, digestive enzyme activity, intestine histology and inflammatory gene expression of silver sillago (*Sillago sihama* Forssk’al) (1775). *Aquacult. Nutr*. 26 1724–1735. 10.1111/anu.13123

[B30] Liu X. H. Ye J. D. Wang K. Kong J. H. Yang W. Zhou L. (2012). Partial replacement of fish meal with peanut meal in practical diets for the Pacific white shrimp, *Litopenaeus vannamei*. *Aquacult. Res*. 43 745–755. 10.1111/j.1365-2109.2011.02883.x

[B31] Naylor R. L. Hardy R. W. Bureau D. P. Chiu A. Elliott M. Farrell A. P. (2009). Feeding aquaculture in an era of finite resources. *Proc. Natl. Acad. Sci*. 106 15103–15110. 10.1073/pnas.0905235106 19805247 PMC2741212

[B32] NRC (2011). *Nutrient Requirements of Fish.* Washington, DC: National Academy Press.

[B33] Parvez S. Raisuddin S. (2005). Protein carbonyls: novel biomarkers of exposure to oxidative stress-inducing pesticides in freshwater fish *Channa punctata* (Bloch). *Environ. Toxicol. Phar*. 20 112–117. 10.1016/j.etap.2004.11.002 21783577

[B34] Roux P. P. Topisirovic I. (2012). Regulation of mRNA translation by signaling pathways. *Cold Spring Harb. Perspect. Biol*. 4 843–853. 10.1128/MCB.00070-18 22888049 PMC3536343

[B35] Shen J. F. Liu H. Y. Tan B. P. Dong X. H. Yang Q. H. Chi S. Y. (2020). Effects of replacement of fishmeal with cottonseed protein concentrate on the growth, intestinal microflora, haematological and antioxidant indices of juvenile golden pompano (*Trachinotus ovatus*). *Aquacult. Nutr*. 26 1119–1130. 10.1111/anu.13069

[B36] Song F. Xu D. D. Mai K. S. Zhou H. H. Xu W. He G. (2016). Comparative study on the cellular and systemic nutrient sensing and intermediary metabolism after partial replacement of fishmeal by meat and bone meal in the diet of turbot (*Scophthalmus maximus* L.). *PLoS One* 11:e0165708. 10.1371/journal.pone.0165708 27802317 PMC5089717

[B37] Su J. Z. Gong Y. L. Mei L. Y. Xi L. W. Chi S. Y. Yang Y. X. (2020). The characteristics of glucose homeostasis in grass carp and chinese longsnout catfish after oral starch administration: A comparative study between herbivorous and carnivorous species of fish. *Br. J. Nutr*. 123 627–641. 10.1017/S0007114519003234 31813383

[B38] Tamura S. Shimomura L. (2005). Contribution of adipose tissue and de novo lipogenesis to nonalcoholic fatty liver disease. *J. Clin. Invest*. 115 1139–1142. 10.1172/JCI24930 15864343 PMC1087181

[B39] Tu Y. Q. Xie S. Q. Han D. Yang Y. X. Jin J. Y. Zhu X. M. (2015). Dietary arginine requirement for gibel carp (*Carassis auratus gibelio* var. CAS III) reduces with fish size from 50 g to 150 g associated with modulation of genes involved in TOR signaling pathway. *Aquaculture* 449 37–47. 10.1016/j.aquaculture.2015.02.031

[B40] Turchini G. M. Trushenski J. T. Glencross B. D. (2018). Thoughts for the Future of Aquaculture Nutrition: Realigning Perspectives to Reflect Contemporary Issues Related to Judicious Use of Marine Resources in Aquafeeds. *N. Am. J. Aquacult.* 81 13–39. 10.1002/naaq.10067

[B41] Wan M. G. Yin P. Fang W. P. Xie S. W. Chen S. J. Tian L. X. (2018). The effect of replacement of fishmeal by concentrated dephenolization cottonseed protein on the growth, body composition, haemolymph indexes and haematological enzyme activities of the pacific white shrimp (*Litopenaeus vannamei*). *Aquacult. Nutr*. 24 1845–1854. 10.1111/anu.12823

[B42] Wang J. Tao Q. Y. Wang Z. Mai K. S. Xu W. Zhang Y. J. (2016). Effects of fish meal replacement by soybean meal with supplementation of functional compound additives on intestinal morphology and microbiome of Japanese seabass (*Lateolabrax japonicus*). *Aquacult. Res*. 48 2186–2197. 10.1111/are.13055

[B43] Wang L. Cui Z. H. Ren X. Li P. Wang Y. (2021). Growth performance, feed cost and environmental impact of largemouth bass *Micropterus salmoides* fed low fish meal diets. *Aquacult. Rep*. 20:100757. 10.1016/j.aqrep.2021.100757

[B44] Yaghoubi M. Torfi M. M. Ghafle M. J. Safari O. Hekmatpour F. Gisbert E. (2020). Lysine and methionine supplementation in high soy protein content diets for silvery-black porgy (*Sparidentex hasta*) juveniles. *Iran. J. Fish. Sci.* 19 1329–1343. 10.22092/IJFS.2019.119235

[B45] Yang B. Y. Zhai G. Gong Y. L. Han D. Yin Z. Xie S. Q. (2018). Different physiological roles of insulin receptors in mediating nutrient metabolism in zebrafish. *Am. J. Physiol.: Endocrinol. Metab.* 315 E38–E51. 10.1152/ajpendo.00227.2017 29351486

[B46] Ye H. Q. Xu M. L. Chen L. L. Tan X. H. Wang A. L. (2019). Effects of dietary plant protein sources influencing hepatic lipid metabolism and hepatocyte apoptosis in hybrid grouper (*Epinephelus lanceolatus*♀× *Epinephelus fuscoguttatus*♂). *Aquaculture* 506 437–444. 10.1016/j.aquaculture.2019.03.075

[B47] Yin B. Liu H. Y. Tan B. P. Dong X. H. Chi S. Y. Yang Q. H. (2018). Cottonseed protein concentrate (CPC) suppresses immune function in different intestinal segments of hybrid grouper ♀*Epinephelus fuscoguttatus*×♂*Epinephelus lanceolatu via* TLR-2/Myd88 signaling pathways. *Fish. Shellfish Immunol*. 81 318–328. 10.1016/j.fsi.2018.07.038 30030116

[B48] Yu H. H. Zhang L. L. Chen P. Liang X. F. Cao A. Z. Han J. (2019). Dietary bile acids enhance growth, and alleviate hepatic fibrosis induced by a high starch diet *via* AKT/FOXO1 and cAMP/AMPK/SREBP1 pathway in *Micropterus salmoides*. *Front.* P*hysiol.* 10:1430–1430. 10.3389/fphys.2019.01430 PMC6882294 31824338

[B49] Yuan X. Y. Jiang G. Z. Cheng H. H. Cao X. F. Shi H. J. Liu W. B. (2019). An evaluation of replacing fish meal with cottonseed meal protein hydrolysate in diet for juvenile blunt snout bream (*Megalobrama amblycephala*): growth, antioxidant, innate immunity and disease resistance. *Aquacult. Nutr*. 25 1334–1344. 10.1111/anu.12954

[B50] Zhang Y. Chen P. Liang X. F. Han J. Wu X. F. Yang Y. H. (2019). Metabolic disorder induces fatty liver in japanese seabass, *Lateolabrax japonicas* fed a full plant protein diet and regulated by cAMP-JNK/NF-kB-caspase signal pathway. *Fish Shellfish Immunol*. 90 223–234. 10.1016/j.fsi.2019.04.060 31029777

[B51] Zhang Y. Xie S. Wei H. Zheng L. Liu Z. Fang H. (2020). High dietary starch impaired growth performance, liver histology and hepatic glucose metabolism of juvenile largemouth bass, *Micropterus salmoides*. *Aquacult. Nutr.* 26 1083–1095. 10.1111/anu.13066

[B52] Zhao W. Liu Z. L. Niu J. (2021). Growth performance, intestinal histomorphology, body composition, hematological and antioxidant parameters of *Oncorhynchus mykiss* were not detrimentally affected by replacement of fish meal with concentrated dephenolization cottonseed protein. *Aquacult. Rep*. 19:100557. 10.1016/j.aqrep.2020.100557

[B53] Zhou Q. L. Habte-Tsion H. M. Ge X. Xie J. Ren M. Liu B. (2018). Graded replacing fishmeal with canola meal in diets affects growth and target of rapamycin pathway gene expression of juvenile blunt snout bream, *Megalobrama amblycephala*. *Aquacult. Nutr*. 24 300–309. 10.1111/anu.12560

